# Characteristics, immunological response & treatment outcomes of HIV-2 compared with HIV-1 & dual infections (HIV 1/ 2) in Mumbai

**Published:** 2010-12

**Authors:** Montaldo Chiara, Zachariah Rony, Mansoor Homa, Varghese Bhanumati, Joanna Ladomirska, M. Manzi, N. Wilson, Deshpande Alaka, A.D. Harries

**Affiliations:** *Medecins sans Frontieres, Mumbai, India*; * *Medecins sans Frontiéres, Medical Department (Operational Research), Operational Centre Brussels, MSF-Luxembourg, Luxembourg*; + *International Union against Tuberculosis & Lung Disease, South East Asia Office, New Delhi, India*; ** *ART Centre, JJ Hospital Mumbai, India*; # *International Union against Tuberculosis & Lung Disease, Paris, France & Tropical Medicine, London, UK*; ## *London School of Hygiene & Tropical Medicine, London, UK*

**Keywords:** ART, HIV-1, HIV-2, Mumbai, treatment outcomes

## Abstract

**Background & objectives:**

Information available on HIV-2 and dual infection (HIV-1/2) is limited. This study was carried out among HIV positive individuals in an urban referral clinic in Khar, Mumbai, India, to report on relative proportions of HIV-1, HIV-2 and HIV-1/2 and baseline characteristics, response to and outcomes on antiretroviral treatment (ART).

**Methods:**

Retrospective analysis of programme data (May 2006-May 2009) at Khar HIV/AIDS clinic at Mumbai, India was done. Three test algorithm was used to diagnose HIV-1 and -2 infection. Standard ART was given to infected individuals. Information was collected on standardized forms.

**Results:**

A total of 524 individuals (male=51%; median age=37 yr) were included in the analysis over a 3 year period (2006-2009) - 489 (93%) with HIV-1, 28 (6%) with HIV-2 and 7(1%) with dual HIV-1/2 infection. HIV-2 individuals were significantly older than HIV-1 individuals (*P*<0.001). A significantly higher proportion of HIV-2 patients and those with dual infections had CD4 counts <200 cells/µl compared to HIV-1. HIV-2 individuals were more likely to present in WHO Clinical Stage 4. Of the 443 patients who were started on ART, 358 (81%) were still alive and on ART, 38 (8.5%) died and 3 were transferred out. CD4 count recovery at 6 and 12 months was satisfactory for HIV-1 and HIV-2 patients on protease inhibitor based regimens while this was significantly lower in HIV-2 individuals receiving 3 nucleoside reverse transcriptase inhibitors.

**Interpretation & conclusions:**

In an urban HIV clinic in Mumbai, India, HIV-2 and dual infections are not uncommon. Adaptation of the current national diagnostic and management protocols to include discriminatory testing for HIV types and providing access to appropriate and effective ART regimens will prevent the development of viral resistance and preserve future therapeutic options.

HIV type I (HIV-1) and type 2 (HIV-2) are very closely related but differ in pathogenicity, natural history and therapy. HIV-1 is more easily transmitted and consequently accounts for the vast majority of global HIV infections. The less transmissible HIV-2 was thought to be largely confined to West Africa (where it is thought to have originated)^1^,^2^ but has spread to parts of Europe and India^3^–^5^.

When compared to HIV-1, HIV-2 infected individuals have a much longer asymptomatic stage, slower progression to AIDS^6^–^8^, slower decline in CD4 count^6^,^9^,^10^ lower mortality^7^,^11^, lower rate of vertical transmission^12^–^14^ and smaller gains in CD4 count in response to antiretroviral treatment (ART)^15^–^17^.

Serologic reactivity to HIV-1 and HIV-2 (HIV-1/2) has also increased in HIV-2 endemic areas over the past decade^18^,^19^. In terms of antiretroviral drug regimens, HIV-2 is intrinsically resistant to non nuclesoside reverse transcriptase inhibitors (NNRTI) such as nevirapine and efavirenz and not all the protease inhibitors (PIs) provide good viral suppression^15^.

Since the introduction of ART, several published papers (particularly from Africa) have described the clinical characteristics, response and treatment outcomes of HIV-1 infected patients on ART^19^–^22^. In contrast, there is a dearth of published information on HIV-2^23^–^24^ (and dual HIV-1/2). Information on the distribution of HIV-2 and dual infection across the Indian subcontinent is limited and current surveillance by the National Programme does not pick up this information^24^. The current national guidelines and the diagnostic and treatment algorithms do not allow for discrimination in the diagnosis of HIV infection by type or provide for treatment alternatives for HIV 2 and dual infected persons. From a public health perspective, it is important to assess the characteristics as well as response to ART for HIV-2 and dual infections.

We therefore carried out a study among HIV positive individuals in an urban referral clinic in Mumbai, India to report on relative proportions of HIV-1, HIV-2 and HIV-1/2, and baseline characteristics, response to and outcomes on ART.

## Material & Methods

### 

#### Study setting and population:

This retrospective study was done between May 2006 and May 2009 at Khar HIV/AIDS clinic in Mumbai, Maharashtra State, India. This clinic is managed by Médecins Sans Frontières (MSF).

MSF started an HIV project in Mumbai in February 2006 in collaboration with the Mumbai district authorities to provide access to populations who did not have access to the public system namely: HIV2, HIV-TB co-infection, Hepatitis B co-infection and drug resistant tuberculosis.

HIV positive patients diagnosed elsewhere are also referred to the MSF clinic where a discriminatory HIV-test is offered free-of charge to identify the type of HIV infection. HIV testing involves a three test algorithm. This includes Microparticle Enzyme Immunoassay (ABBOTT-AXSYM, Mumbai, India), Enzyme Immunoassay (MICROELISA-J Mitra, Mumbai, India) and Rapid Immunochromatography (TriDot, Mumbai, India). Tridot discriminates between HIV-1 and HIV-2. Confirmatory Western blot is done in the MSF Clinic for all the samples positive for HIV-2 or -1 and 2. All HIV-positive individuals undergo a full clinical and immunological assessment.

The study population for determining relative proportions of HIV types included all consecutive people living with HIV (PLHA) registered in the MSF clinic, while response to ART and treatment outcomes were assessed among all consecutive patients who started ART. The Khar clinic is open to all patients in Mumbai irrespective of geographical residence.

#### Antiretroviral therapy:

Apart from pregnant women, HIV-positive individuals were eligible for ART if they presented in WHO Clinical Stage 3 (with CD4 count equal or less than 350 cells/µl), WHO stage 4 irrespective of CD4 count or have a CD4-lymphocyte count <200 cells/µl (irrespective of WHO stage^2^,^5^. HIV-infected pregnant women were initiated on ART when their CD4-lymphocyte count was ≤350 cells/µl, regardless of clinical stage. Once started on ART patients were reviewed by a clinician 2 wk later and monthly thereafter. Individuals who started ART had their WHO clinical stage, CD4 cell count and weight measured at baseline and then at 6 monthly intervals.

For HIV-1, first-line ART was a fixed dose combination of stavudine (d4T) or zidovudine (AZT), lamuvudine (3TC) and nevirapine (NVP) or efavirenz (EFV). In case of double toxicity to zidovudine (AZT) and stavudine (d4T), the alternative treatment is tenofovir (TDF). In the event of renal failure (creatinine clearance <50) tenofovir (TDF) was replaced with abacavir (ABC).

For HIV-2, first-line ART was selected according to the background (previous history of intake) of ART. For ART naïve patients, 3 nucloside reverse transcriptase inhibitors (NRTIs) were given: TDF and a fixed dose combination of zidovudine (AZT) and lamivudine (3TC). In case of renal failure, TDF was replaced with ABC.

For ART experienced patients, a PI based regimen was given which included indinavir (IDV), ritonavir (RTV) and fixed dose combination of stavudine (d4T) and lamuvudine (3TC). Boosted indinavir was preferred to lopinavir/ritonavir for HIV-2 infection based on previous information^15^,^26^–^29^. Lopinavir/ritonavir (LPV/r) was given only in case of severe intolerance to IDV. For HIV-2 and HIV-1&2 co-infection, the first line treatment was a fixed dose combination of d4T and 3TC plus lopinavir/ritonavir (LPV/r), in the heat stable form. Lopinavir/ritonavir (LPV/r) in this case is preferred to indinavir because the disease is driven by HIV-1 infection.

#### Data collection and treatment outcomes:

Structured forms were used to gather information on HIV status, HIV-type, WHO clinical stage and CD4 cell count at presentation. ART treatment outcomes were recorded on a monthly basis from Treatment cards. Data were entered and stored in the FUCHIA database system (FUCHIA, Epicentre, Paris, France). Data were collated on all patients from May 2006 and were censored at the end of June 2009.

Standardised treatment outcomes were monitored on a monthly basis and were defined as: alive and on ART (patient alive and on ART at the facility where he/she was registered); died (patient who had died for any reason while on ART), lost to follow up (patient who did not attend the clinic for one month or more after their scheduled follow up appointment); stopped treatment (patient known to have stopped treatment for any reason during treatment); transferred out (patient who had transferred-out permanently to another treatment facility).

#### Statistical analysis:

Previous reports from India^3^,^4^ have showed a HIV-2 prevalence of about 5 per cent. Assuming that the same proportion is applicable to our setting and the worst possible result that we would like to detect is 7 with a 95 per cent confidence interval and 2 per cent error, a total of at least 456 consecutive individuals was required.

Differences between groups were compared using the χ^2^ test for categorical variables and the Wilcoxon rank-sum test for continuous variables. The level of significance was set at *P*<0.05. Data were analysed using the STATA/IC 10.0 software (Stata corporation, Texas 77845, USA).

This study received ethical clearance from the ethics review committees of the international Union Against TB and Lung disease and Médecins Sans Frontières.

### Results

#### Cohort characteristics:

A total of 611 individuals tested HIV-positive, of whom 87 did not have HIV-type specified and were therefore, excluded from the analysis. Among the remaining 524 individuals included in the study, 272 (52%) were male, (median age 37 yr, range: 2-65 yr). Nearly half (43%) reported earning a regular income, 96 (18%) were housewives, 33 (6%) were sex workers, 12 (2%) were students, 3 (1%) were businessmen and 156 (30%) were unemployed (Table I).

**Table I:** Characteristics of individuals with HIV-1, HIV-2 and HIV-1/2 in MSF Khar Clinic (n=524)

Variable	HIV-1 n (%)	HIV-2 n (%)	*P* value^a^^b^	HIV-1&2 n (%)	*P* value^a^^c^
Total	489 (93)	28 (6)	-	7 (1)	-
Sex					
Females	210 (43)	13 (46)	0.7	2 (29)	0.7
Males	252 (51)	15 (54)		5 (71)	
Transgender	27 (6)	0	-	0	-
Age (yr)					
< 15	36 (8)	1 (4)	0.6	0 (0)	-
15-29	96 (20)	1 (4)	0.03	1 (14)	0.92
30-44	293 (60)	14 (50)	0.29	6 (86)	0.3
45+	64 (12)	12 (42)	<0.001	0 (0)	-
Age, yr, median (IQR)	33 (31-42)	44 (40-48)	<0.001	39 (31-43)	0.37
Marital status					
Single	110 (22)	6 (21)	0.9	1 (14)	0.9
Married	277 (57)	17 (61)	0.7	4 (58)	0.7
Widowed	88 (18)	5 (18)	0.9	2 (28)	0.8
Divorced/separated	14 (3)	0 (0)	-	0 (0)	-
Baseline CD4 cell count (cell/µl)					
< 50	25 (5)	2 (7)	0.93	0)	-
50-199	165 (34)	21 (75)	<0.001	6 (86)	0.01
200-349	232 (47)	1 (4)	<0.001	1 (14)	0.1
≥350	67 (14)	4 (14)	0.8	0	-
Median (IQR) (n=5161)	221(76-307)	143 (73-144)	0.08	127 (91-160)	0.3
WHO clinical stage					
I	151 (31)	0 (0)	-	2 (29)	0.7
II	83 (17)	4 (14)	0.9	0 (0)	-
III	183 (37)	11 (39)	0.8	2 (29)	0.9
IV	72 (15)	13 (47)	<0.001	3 (43)	0.1

IQR, interquartile range; ART, antiretroviral therapy; WHO, World Health Organization;

a χ^2^ test for categorical variables and Wilcoxon ranksum test for continuous variables;

b Comparing HIV-1 with HIV- 2;

c Comparing HIV-1 with HIV-1 & 2

There were 489 (93%) individuals with HIV-1, 28 (6%) with HIV-2 and 7 (1%) with dual HIV-1/2 infection. HIV-2 individuals were significantly older (> 45 yr) than HIV individuals. A significantly higher proportion of HIV-2 patients and those with dual infections had CD4 counts <200 cells/µl compared to HIV-1. Among HIV-1, HIV-2 and HIV-1/ 2 infections, 414 (85%), 25 (89%) and 4 (57%) started ART respectively.

#### Characteristics of patients on ART and immunologic outcomes:

Among the 524 HIV-positive individuals with HIV-type classified, 443 started ART. Of these, 414 (93%) had HIV-1, 25 (6%) had HIV-2 and 4 (1%), HIV-1/2. Contrary to the trend seen at baseline, HIV-1 patients were generally older at ART initiation compared with HIV-2 patients. Ten (40%) HIV-2 patients had been started on a regimen of 3 NRTI and 15 (60%) started on a regimen of 2NRTIs and 1 PI (Table II).

**Table II:** Characteristics and treatment outcomes of patients on ART (>6 months) according to HIV type in Khar Clinic (n=443)

Variable	HIV-1^a^ n (%)	HIV-2 n (%)	*P* value^b^^c^	HIV-1&2 n (%)	*P* value^b^^d^
Total	414	25	-	4	-
Sex					
Females	231 (56)	11 (44)	0.2	0 (0)	-
Males	164 (40)	14 (66)	0.01	4 (100)	0.2
TG	19 (4)	0		0	-
Age (yr)					
<15	18 (4)	0 (0)	-	0 (0)	
15-29	78 (19)	15 (60)	<0.001	4 (100)	
30-44	274 (66)	10 (40)	<0.001	0 (0)	
45+	44 (11)				
Age, yr, median (IQR)	37 (33-43)	45 (41-49)	0.01	44 (39-43)	0.15
WHO clinical stage					
Stage I or II (CD4<250 cells/µl)	185 (45)	5 (20)	0.01	0 (0)	-
Stage III	128 (31)	12 (48)	0.07	2 (50)	0.7
Stage IV	101 (21)	8 (32)	0.19	2 (50)	0.8
CD4 count, (cells/µl)					
<50	73 (18)	3 (12)	0.6	0 (0)	-
50-199	244 (59)	21 (84)	0.01	4 (100))	0.2
200-349	87 (21)	1 (4)	0.03	0 (0)	-
≥350	10 (2)	0 (0)	-	0 (0)	-
Median (IQR)	189 (72-262)	96 (73-111)	0.03	114 (79-150)	0.4
ART regimen					
2 NRTIs + 1 NNRTI	334 (81)	0 (0)	-	0 (0)	
3 NRTIs	5 (1)	10 (40)	< 0.001	0 (0)	
2 NRTIs + Pls	75 (18)	15 (60)	< 0.001	4 (100)	0.8
ART outcomes					
Alive on ART	333 (80)	25 (100)	-	2 (50)	-
Dead	36 (9)	0 (0)		2 (50)	
Lost to follow up	31 (8)	0 (0)		0 (0)	
Transferred out	14 (3)	0 (0)		0 (0)	

IQR, interquartile range; ART, antiretroviral therapy; WHO, World Health Organization; NRTI, nucleoside reverse transcriptase inhibitors;NFV, nelfinavir; LPV/r, lopinavir/ritonavir; IDV, indinavir; NNRTI, non-nucleoside reverse transcriptase inhibitors;

a 1unknown outcome for HIV-1;

b X^2^ test for categorical variables and Wilcoxon rank-sum test for continuous variables;

c Comparing HIV-1 with HIV- 2;

d Comparing HIV-1with HIV-1 & 2

The mean increase in CD4 for HIV-1 was 195, not statistically different from the one observed in HIV-2 patients treated with a PI-based regimen at 6 and 12 months (Fig.).

**Fig.**
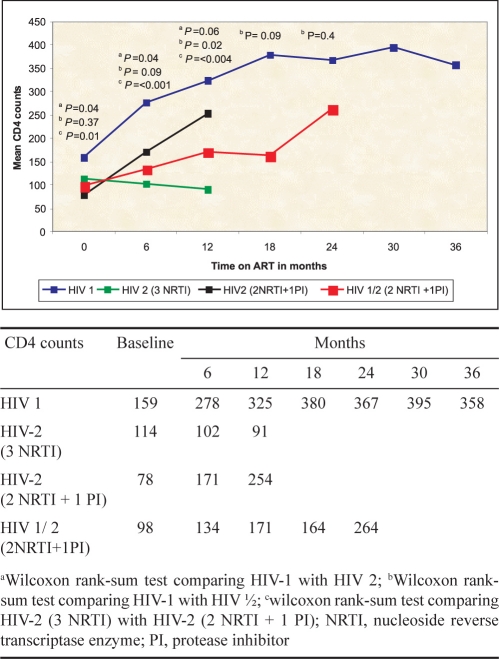
Mean CD4 cell counts in patients on ART in Mumbai during follow up according to HIV type and ARV regimen.

The immunological results obtained with a PI-based regimen were satisfactory both in naïve and non naïve patients. A slower but constant CD4 gain was observed in the patients with dual infection at 6 and 12 months. In contrast, a decline in CD4 count was seen in the group of HIV-2 patients treated with 3 NRTIs. In view of this decline, these patients were all switched subsequently to a PI based regimen.

Treatment outcomes censored at the end of June 2009 included: 358 (81%) alive and on ART, 38 (9%) deaths, 31 (7%) lost to follow up and 14 (3%) transferred out (Table II).

## Discussion

This is one of the first reports describing baseline characteristics and response to ART among patients with HIV-1, HIV-2 and dual HIV-1/2 in the routine setting of an HIV Clinic in Mumbai, India. Of particular relevance is the finding that HIV-2 and dual infections are not uncommon, and it is likely that the situation is similar in other clinics in the same setting. It is necessary that the Ministry of Health along with partners works towards revision and adaptation of the current diagnostic and management protocols for HIV. What is urgently needed is to (*i*) include discriminatory testing and diagnosis of all HIV types; (*ii*) increase access to discriminatory HIV-1 and HIV-2 test kits at HIV testing sites. Patients found to have dual infections should also have the possibility for access to confirmatory HIV type-testing at identified referral laboratories; and (*iii*) provide access to effective first-line ART regimens for both HIV-1, HIV-2 and dual infections in order to avoid the development of viral resistance that will compromise future therapeutic options particularly for HIV-2.

Surprisingly HIV-2 individuals presented with lower CD4 counts and a significantly higher proportion were in WHO Clinical Stage 4 when compared to HIV-1. This probably reflects delays in diagnosis which can be due to a slower progression of the disease or to a lack of systematic screening for HIV-2. In the absence of discriminatory HIV testing, HIV-2 individuals might be assumed to have HIV-1 and be wrongly placed on an ineffective ART regimen and progress in their illness before presenting to our clinic. Misdiagnosis of HIV-1/2 may also be a problem in many peripheral centres in Mumbai and accurate identification of dual HIV-1/2 infection remains a diagnostic challenge. Although limited by sample size, two (50%) of the four patients with dual infections died during ART and this might be related to late diagnosis and ineffective initial ART regimens. This is suggested as both patients were non naïve and had very low baseline CD4 counts. Discriminating between HIV-1 and HIV-2 is relatively simple as the rapid HIV detection assays (if available) are sensitive and specific for HIV-1 and HIV-2. However, these assays lack specificity for dual HIV-1/2 infection. Different studies have shown that among individuals diagnosed as dual seropositive, often only HIV-1 or HIV-2 DNA is isolated (more often HIV-1), a phenomenon commonly thought to be explained by cross-reactivity^17^,^30^,^31^. However, this has important management implications and all patients found with dual infections should ideally undergo confirmatory testing using polymerase chain reaction techniques or Western blot. These are currently not available in the government ART centres.

Despite the fact that baseline CD4 cell counts at ART initiation were relatively similar between the different serotypes, CD4 cell recovery appeared to be poorer for dual infected patients than for HIV-1 and HIV-2, at 6 and at 12 months. The trend in CD4 gain in HIV-2 patients who were on a PI regimen was encouraging considering that all these patients were ART exposed (previously exposed to NNRTIs based regimens). The reason for this can be the systematic use of indinavir as the standard PI for HIV-2 in our setting^22^.

The failure of a 3NRTI regimen in terms of immunological recovery in our cohort was quite significant and highlights the need to review treatment options for HIV-2 infection. Considering that all HIV-2 patients who started 3 NRTIs were NNRTI naïve, the failure could be due to an infection with non-naïve virus (primary resistance), to undocumented previous exposure to double NRTIs (an ineffective first-line ART regimen containing an NNRTI) or due to an intrinsic incomplete suppression of viral loads on the triple NRTI regimen. This observation persuaded us to switch all the patients to a superior PI based regimen, and in future this will be the regimen of choice.

The strengths of this study are that: (*i*) loss to follow up was relatively low (7%), and (*ii*) the data came from a programme setting and were likely to reflect the operational reality on the ground. The limitations of the study included (*i*) limited number of patients and thus limited statistical power to detect statistically significant differences, (*ii*) a relatively short period of observation: the cohort thus needed to be followed up for a longer time period; and (*iii*) there were no data on viral load either at the start or during therapy.

In conclusion, in an HIV clinic in Mumbai, India, HIV-2 and dual infections are not uncommon and it is likely that the situation is similar in other clinics. An HIV testing strategy that discriminates between HIV types and access to effective first-line ART regimens must be considered to manage these patients effectively.
